# Chemical composition of the *Kaempferia galanga* L. essential oil and its *in vitro* and *in vivo* antioxidant activities

**DOI:** 10.3389/fnut.2023.1080487

**Published:** 2023-02-02

**Authors:** Si-Yu Wang, Lei Cai, Na Yang, Fang-Fang Xu, Yun-Shan Wu, Bo Liu

**Affiliations:** ^1^The Second Clinical Medical College, Guangdong Provincial Key Laboratory of Clinical Research on Traditional Chinese Medicine Syndrome, Guangzhou University of Chinese Medicine, Guangzhou, China; ^2^Guangdong Provincial Key Laboratory of Laboratory Animals, Guangdong Laboratory Animals Monitoring Institute, Guangzhou, China; ^3^School of Pharmaceutical Sciences, Guangzhou University of Chinese Medicine, Guangzhou, China; ^4^Guangzhou Key Laboratory of Chirality Research on Active Components of Traditional Chinese Medicine, Guangzhou, China; ^5^State Key Laboratory of Dampness Syndrome of Chinese Medicine, Guangzhou, China

**Keywords:** *Kaempferia galanga* L., essential oil, antioxidant activity, zebrafish, oxidative stress

## Abstract

**Introduction:**

Oxidative stress is closely related to the development of many diseases. Essential oils (EOs) show potent antioxidant activity from natural sources. *Kaempferia galanga L*. is an important medicine rich in high-value essential oil (KGEO). However, the antioxidant activity of KGEO remains to be fully studied.

**Methods:**

Chemical composition of KGEO was analyzed using gas chromatography-mass spectrometry (GC-MS). The antioxidant activity was determined using the DPPH, ABTS, hydroxyl radical scavenging assays and reducing power assay *in vitro*. A zebrafish model was used to evaluate the protective effect of KGEO against H2O2-induced oxidative stress damage *in vivo*.

**Results:**

The major components of KGEO were found to be *trans* ethyl p-methoxycinnamate (32.01%), n-pentadecane (29.14%) and *trans* ethyl cinnamate (19.50%). *In vitro* pharmacological results showed that KGEO had good free radical scavenging capacity in DPPH, ABTS, and hydroxyl radical scavenging assays (IC_50_ values: 19.77 ± 1.28, 1.41 ± 0.01, and 3.09 ± 0.34 mg/mL, respectively) and weak reducing capacity in the reducing power assay (EC_50_ value: 389.38 ± 4.07 mg/mL). *In vivo* zebrafish experiments results indicated that the survival rate and heart rate increased, and ROS generation, cell death, and lipid peroxidation were attenuated after KGEO treatment. In addition, a decrease in malondialdehyde (MDA) levels and increases in superoxide dismutase (SOD), catalase (CAT) and glutathione peroxidase (GSH-Px) activities were observed in the KGEO-treated groups.

**Discussion:**

This study validated the *in vitro* and *in vivo* antioxidant activities of KGEO, which provides a theoretical basis for a profound study of KGEO and its application in the pharmaceutical, food and cosmetic industries.

## Graphical Abstract

The chemical composition of KGEO was determined by using GC-MS. The antioxidant activities of KGEO were evaluated through various experiments *in vitro* and *in vivo*. 
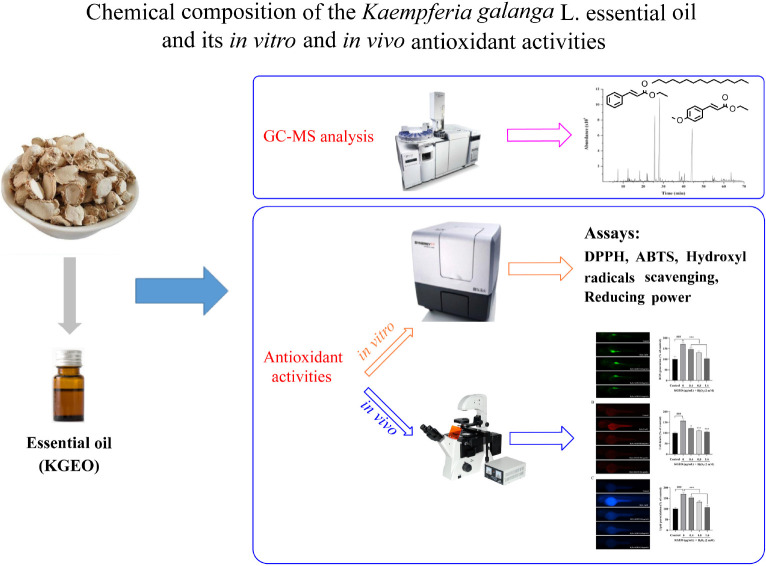

## 1. Introduction

Oxidative stress is associated with the development of many diseases, such as cancer, diabetes, aging, inflammation, and Parkinson’s disease (1). Oxidative stress is induced due to an imbalance between reactive oxygen species (ROS) and antioxidants in the human body (2–4). Normally, the production and clearance of ROS such as hydrogen peroxide (H_2_O_2_), hydroxyl radicals (OH⋅), and superoxide anion radicals (O_2_⋅^–^) are balanced in the processes of normal cellular metabolism and the antioxidant system (2). However, abnormal ROS production caused by excessive environmental stimulation results in oxidative stress and related diseases (3), and the accumulation of excessive ROS causes irreversible oxidative damage (4). Synthetic antioxidants have been used to inhibit the oxidative damage caused by the generation of excessive ROS. However, they are suspected to have side effects, such as digestive problems. Thus, new natural antioxidants without toxicity have become the focus of numerous studies.

Essential oils (EOs) are an important class of compounds with diverse pharmacological activities, including antioxidant, antimicrobial, and anti-inflammatory activities. They are extracted from plant materials and are mainly composed of monoterpenes and sesquiterpenes (5–6). *Kaempferia galanga* is an important herbal medicine and is widely used in India, China, Myanmar, Bangladesh, and Thailand (7). It has been used in folk medicine to treat indigestion, rheumatism, toothaches, asthma, piles, and wounds (8). Pharmacological studies have shown that *K. galanga* possesses antimicrobial (9), antioxidant (10), anti-inflammatory and analgesic (11), vasorelaxant (12), and antitumour (13) activities. As the main component of *K. galanga*, *K. galanga* essential oil (KGEO) also plays an important medicinal role and is used to treat indigestion, rheumatism, asthma, coughs, and skin issues (8).

Excessive free radicals induce oxidative stress, which is harmful to the human body (14). Novel antioxidants from plants and their underlying mechanisms have attracted much attention because of the side effects of synthetic drugs (15). As a free radical scavenger, EO can prevent various types of damage to the body caused by free radicals.

However, the antioxidant activity of KGEO remains to be fully studied. The aim of the study was to investigate the chemical composition characteristics of KGEO by using gas chromatography-mass spectrometry (GC-MS). Furthermore, the antioxidant activity of KGEO were systematically evaluated *in vitro* and *in vivo*. The findings of this study indicated its potential to develop as novel antioxidant in the pharmaceutical, food, and cosmetic industries.

## 2. Materials and methods

### 2.1. Materials

#### 2.1.1. Plant material

*Kaempferia galanga* rhizomes were purchased from Qingping Chinese medicinal herbs market (Guangdong Province, China), and were identified by deputy chief pharmacist Junbiao Wu (The Second Clinical Medical College, Guangzhou University of Chinese Medicine, Guangzhou, China). Prior to use, the rhizomes were ground into powder.

#### 2.1.2. Solvents and chemicals

The reference standard of n-alkanes (C_8_−C_20_) was received from ANPEL Laboratory Technologies (Shanghai) Inc. (Shanghai, China). 2,2-diphenyl-1-picrylhydrazyl (DPPH, 98%), 2,2′-amino-di(2-ethyl-benzothiazoline sulfonic acid-6) ammonium salt (ABTS, 98%), ascorbic acid (≥ 99%), 2′,7′-dichlorodihydrofluorescein diacetate (DCF-DA), and acridine orange were acquired from Sigma-Aldrich (Shanghai, China). Malondialdehyde (MDA), superoxide dismutase (SOD), catalase (CAT), and glutathione peroxidase (GSH-Px) were obtained from Nanjing Jiancheng Biotechnology Co., Ltd (Nanjing, China). Dimethyl sulfoxide (DMSO) was obtained from MP Biomedicals (CA, USA). Diphenyl-1-pyrenylphosphine (DPPP, catalog no. DJ799) was obtained from Macklin (Shanghai, China). Other chemicals used in this study were of analytical grade.

### 2.2. Essential oil extraction

A Clevenger-type apparatus with ethanol was used to extract the EO of the rhizome powder (500 g) by distillation for 6 h, and then water was removed with anhydrous sodium sulfate. KGEO was stored in a well-closed container at 4°C.

### 2.3. GC-MS analysis

The volatile compounds were identified by GC-MS (Agilent 7890A-5975C, Agilent Technologies, USA) using an HP−5MS capillary column (30 m × 0.25 mm, film thickness 0.25 μm). Helium was the carrier gas at 1.0 ml/min. One microlitre was injected with a split ratio of 1/10, and the injector temperature was 250°C. The oven temperature was increased from 60 to 122°C at 3°C/min, subsequently raised to 126, 148, 152, and 250°C at 0.5, 2, 0.5, and 5°C/min, respectively, and held at 250°C for 2 min. The mass spectrometry (MS) parameters were as follows: ion source temperature, 230°C; electron impact, 70 eV; scan mass range, 50−550 m/z (1.0 scan/s). The retention indices (RIs) of all components were calculated by using n-alkanes (C_8_−C_20_) under the same conditions as those of the normal sample to identify the constituents of the EO by comparing the RI of the n-alkanes and the spectra with the MS Library (NIST 14) under identical experimental conditions.

### 2.4. *In vitro* antioxidant activity

#### 2.4.1. DPPH radical scavenging activity

The DPPH assay was used to determine the DPPH radical scavenging activity of KGEO according to the described method (16–17) after making the changes. One milligram of DPPH was dissolved in ethanol (5 ml) to prepare a fresh DPPH working solution. Fifty microlitres of different concentrations of KGEO sample solutions (1, 5, 10, 20, 30, and 40 mg/ml) were mixed well with 50 μL of 0.5 mM DPPH solution to react in the dark at room temperature for 30 min as a sample group. For the blank group, the sample was replaced with an equal volume (50 μL) of ethanol, and ascorbic acid was used as a positive control. A microplate reader (M1000pro, Tecan, Männedorf, Switzerland) was used to determine the absorbance at 517 nm. Three replicates of the experiment were carried out. The IC_50_ (mg/ml) value was determined by the following Eq. 1:


(1) $$Scavengingrate\left(\%\right)=\frac{A_{0}-A_{1}}{A_{0}}\times 100\%$$

where *A*_0_ represents the absorbance values of the control and *A*_1_ represents the absorbance values of KGEO. The scavenging rate and IC_50_ of ascorbic acid were calculated under the same conditions.

#### 2.4.2. ABTS radical scavenging activity

The ABTS assay was carried out based on a previously reported method (16) with some modifications. To prepare the ABTS radical stock solution, ABTS (7.4 mM) solution and K_2_S_2_O_8_ (2.45 mM) solution were mixed with an equal volume and reacted for 12−16 h in the dark. Before use, the ABTS radical stock solution was diluted withphosphate buffered saline (PBS) until the absorbance was 0.70 ± 0.02 at 734 nm. Fifty microlitres of different concentrations of sample solutions (0.4, 0.6, 0.8, 1.0, 1.2, 1.4, 1.6, 1.8, and 2.0 mg/ml) were mixed with 150 μL of ABTS radical working solution to react for 6 min in the dark at room temperature, which was used as the experimental group. For the blank group, the sample solutions were replaced by the same volume of ethanol (50 μL). The absorbance of each group was measured at 734 nm using a microplate reader. The results are presented as the IC_50_ value (mg/ml), and the scavenging rate was determined according to Eq. 1. The inhibition percentage and IC_50_ of ascorbic acid were calculated under the same conditions.

#### 2.4.3. Hydroxyl radical scavenging activity

The ability of KGEO to inhibit hydroxyl radicals was evaluated according to a reported method (18) with some modifications. KGEO samples (1 ml) with a range of concentrations (1, 5, 10, 20, and 30 mg/ml) were combined with 1 ml of 1 mM salicylic acid ethanol solution, 1 ml of FeSO_4_ (1 mM), and 1 ml of 0.01% H_2_O_2_ (v/v), which were mixed in order. The mixture was maintained at 37°C for 30 min, and then the absorbance (*A*_*i*_) was measured at 510 nm. Ascorbic acid was used for the positive control group. An equal volume of distilled water replaced the 0.01% H_2_O_2_ to prepare *A*_*j*_. *A*_0_ was prepared with an ethanol solution instead of KGEO samples. The ability of KGEO to inhibit hydroxyl radicals was determined using Eq. 2:


(2) $$Scavengingrate\left(\%\right)=\frac{A_{0}-\left(A_{i}-A_{j}\right)}{A_{0}}\times 100\%$$

#### 2.4.4. Reducing power assay

The total reduction ability of KGEO was evaluated by using the method reported in the literature (19–20) with some modifications. KGEO samples (0.5 ml) of a range of concentrations (0.2, 0.3, 0.4, 0.5, 0.6, and 0.7 g/ml) were mixed with 0.2 M phosphate buffer (pH 6.6) solution (2.5 ml) and 1% potassium ferricyanide (2.5 ml) in order. Then, the mixture was maintained at 50°C for 20 min. Afterward, 10% trichloroacetic acid (2.5 ml) was added, followed by incubation for 10 min at room temperature. After incubation, 0.10% ferric chloride (0.5 ml) and distilled water (2.5 ml) were added to the mixture (2.5 ml), and then the absorbance was measured at 700 nm. The EC_50_ value was the value at which the absorbance was 0.5. Ascorbic acid was used as the positive control.

### 2.5. *In vivo* antioxidant activity

#### 2.5.1. Zebrafish husbandry

Wild-type AB zebrafish were provided by Guangdong Laboratory Animals Monitoring Institute. The zebrafish were kept at 28.5 ± 0.5°C with a 14:10 h light/dark cycle and fed flake food three times daily. Embryos were obtained from natural spawning, collected after 45 min of light, and then incubated in E3 medium (21) (CaCl_2_ 0.33 mM, NaCl 5 mM, KCl 0.18 mM, MgSO_4_ 0.34 mM).

#### 2.5.2. Waterborne exposure of embryos to KGEO and H_2_O_2_

We evaluated the effect of KGEO against H_2_O_2_-induced embryotoxicity. KGEO was dissolved in DMSO. The 48 h post-fertilization (hpf) embryos were treated with different concentrations of KGEO (0.4, 0.8, and 1.6 μg/ml) for 1 h. After 1 h, H_2_O_2_ (2 mM) was added as a stimulant. DMSO (0.32%) was used as the solvent control. The embryos were rinsed in fresh medium, and the survival rate was examined after 24 h.

#### 2.5.3. Heartbeat rates of zebrafish embryos

The heartbeat rates of zebrafish embryos were measured after 24 h of H_2_O_2_ (2 mM) stimulation at 72 hpf. The average heart rate of zebrafish embryos was measured by manual counting with a 10 s period for 15 live zebrafish embryos under a microscope (Nikon Ci-E, Japan). The heartbeat rates were expressed as a percentage.

#### 2.5.4. Protective effect of KGEO against H_2_O_2_-induced oxidative stress in zebrafish embryos

Intracellular ROS, cell death, and lipid peroxidation were measured by DCF-DA, acridine orange, and DPPP assays to assess the protective effect of KGEO against H_2_O_2_-induced oxidative stress (22). The zebrafish embryos were treated with phenylthiourea (2 mg/ml) to inhibit pigment pattern formation in zebrafish for better observation of fluorescence images starting at 5 hpf. The 48 hpf zebrafish embryos were treated with or without KGEO (0.4, 0.8, and 1.6 μg/ml) for 1 h. Then, H_2_O_2_ (2 mM) was added, followed by incubation for 24 h. At 72 hpf, the embryos were washed with E3 medium and transferred into 24-well plates. DCF-DA is an oxidation-sensitive fluorescent probe dye used to detect ROS levels. The zebrafish embryos were incubated with DCF-DA solution (2.5 μM) for 50 min at 28°C in the dark. Apoptotic cells were detected in embryos by using acridine orange (7 μg/ml), a nucleic acid-selective fluorescent dye, and the embryos were incubated for 30 min in the dark at 28°C. When DPPP is oxidized, it becomes fluorescent. DPPP is a fluorescent probe used to measure lipid peroxidation for the detection of membrane damage. The embryos were treated with DPPP solution (20 μg/ml) and incubated for 40 min in the dark at 28.5°C. After incubation, the embryos were washed with E3 medium at least three times and anesthetized with MS222 (150 μg/ml) before visualization. Fluorescence images of embryos were captured using a fluorescence microscope (Nikon Ci-E, Japan), and the fluorescence intensity of individual embryos was quantified using ImageJ software (Fiji version) (National Institutes of Health, Bethesda, USA).

#### 2.5.5. Determination of antioxidant enzyme activities and malondialdehyde levels in zebrafish embryos

The 48 hpf zebrafish embryos were treated with or without KGEO (0.4, 0.8, and 1.6 μg/ml) for 1 h and then incubated with H_2_O_2_ (2 mM) for 24 h. At 72 hpf, the embryos from each group were collected and homogenized with buffer (pH 7.2) containing EDTA-2Na (0.0001 M), Tris−HCl (0.01 M), and NaCl (0.65%) using a tissue homogenizer (1,700 r/min, 30 s × 3). After centrifugation (3,000 r/min, 15 min), the supernatants were collected immediately to determine the activity of SOD, CAT and GSH-Px and MDA levels according to the manufacturer’s directions (A003−1, A001−1, A007−1, and A005, Jiancheng, Nanjing, China).

### 2.6. Statistical analysis

Data were analyzed using SPSS 26.0 (SPSS Software, Chicago, IL, USA). One-way ANOVA followed by least significant difference (LSD) or Dunnett’s multiple comparisons test was employed to analyze the statistical significance between the control group and other treatment groups. The non-parametric data were analyzed by a Kruskal−Wallis test. Data are presented as the means ± SDs of three independent experiments. *P* < 0.05 was considered statistically significant (^###^*P* < 0.001 compared with the H_2_O_2_ group; *P < 0.05, ^**^P < 0.01, and ^***^P < 0.001 compared with the control group).

## 3. Results

### 3.1. Chemical composition of *K. galanga* essential oil

The KGEO chemical components that were determined are shown in Table 1, which are arranged in the order of elution from the HP−5MS column. Twenty-eight constituents were found, which comprised 95.18% of the KGEO. Phenylpropanoids (53.60%) were the dominant class in the EO, followed by hydrocarbons (33.18%), monoterpenoids (4.07%), and sesquiterpenoids (2.20%). The most abundant chemical compounds were determined to be *trans* ethyl p-methoxycinnamate (32.01%), n-pentadecane (29.14%), and *trans* ethyl cinnamate (19.50%), which together represented 80.65% of the EO.

**TABLE 1:** Chemical composition of the *K. galanga* essential oil.

No.	Compound name	RI^a^	RI^b^	Composition (%)
1	Eucalyptol	1031	1031	0.49
2	p-Mentha-1,5-dien-8-ol	1164	1166	0.24
3	endo-Borneol	1169	1168	1.02
4	2-Caren-4-ol	1179	1181	0.36
5	p-Cymen-8-ol	1190	1188	0.24
6	Eucarvone	1252	1245	0.21
7	Benzaldehyde, 4-methoxy-	1256	1258	0.40
8	3-Caren-5-one	1312	1314	1.31
9	3-Carene-2,5-dione	1324	1324	0.44
10	Anisaldehyde dimethyl acetal	1368	-	0.43
11	Cyperene	1398	1398	0.73
12	Tetradecane	1402	1403	0.75
13	(-)-α-gurjunene	1407	1408	0.14
14	*trans* ethyl cinnamate	1467	1467	19.50
15	n-Pentadecane	1507	1500	29.14
16	γ-cadinene	1514	1514	0.81
17	Germacrene B	1553	1550	0.28
18	Humulene 6,7-epoxide	1603	1606	0.24
19	Ethyl 4-methoxycinnamate	1662	1669.7	2.09
20	6,9-Heptadecadiene	1670	1675	0.49
21	8-Heptadecene	1678	1695	1.06
22	Heptadecane	1702	1711	1.51
23	*trans* ethyl p-methoxycinnamate	1758	1760	32.01
24	n-Pentadecanol	1780	1775	0.23
25	Dibutyl phthalate	1966	1965	0.23
26	Ethyl 3-(3,4-dimethoxyphenyl)acrylate	1970	1968	0.38
27	Hexadecanoic acid, ethyl ester	1999	1996	0.16
28	Linoleic acid ethyl ester	2167	2163	0.29
	Total identified			95.18
	Monoterpenoids			4.07
	Sesquiterpenoids			2.2
	Phenylpropanoids			53.6
	Hydrocarbons			33.18
	Aliphatic acid esters			0.45
	Others			1.68

^a^Retention index of n-alkanes (C_8_-C_20_) on a capillary HP-5MS column.

^b^Literature retention indices.

### 3.2. *In vitro* antioxidant activity of *K. galanga* essential oil

Because of the complex reactive facets of EO, at least two antioxidant systems should be employed to ensure the reliability of its antioxidant activity. Therefore, four complementary spectrophotometric methods, DPPH, ABTS, hydroxyl radical scavenging and reducing power assays, were used to carry out the following experiments. The results are presented in Table 2.

**TABLE 2:** Antioxidant capacity of the *K. galanga* essential oil measured by different assays.

Samples	DPPH (EC)	ABTS (IC_50_)	Hydroxyl radical (IC_50_)	Reducing power (EC_50_)
Essential oil (mg/ml)	19.77 ± 1.28	1.41 ± 0.01	3.09 ± 0.34	389.38 ± 4.07
Ascorbic acid (mg/ml)	0.03 ± 0.38	0.02 ± 0.08	0.51 ± 16.34	0.18 ± 1.88

Each value is expressed as the mean l’ standard deviation (*n* = 3).

IC_50_, inhibition concentration 50%; EC_50_, effective concentration at which the absorbance was 0.5.

#### 3.2.1. DPPH radical scavenging activity

DPPH is often used in antioxidant experiments as a stable radical (23). The ability of KGEO to scavenge DPPH radicals was studied. In the oxidation reaction, KGEO showed a potential DPPH radical scavenging effect with an IC_50_ value of 19.77 ± 1.28 mg/ml (Table 2), with an increase in DPPH radical scavenging with increasing KGEO concentration. The scavenging effect of KGEO at a concentration of 10 mg/ml was 52.70%, and the activity appeared to be dose-dependent (24). This may be related to the plant source and processing procedure based on the content of its main components. DPPH radicals can be scavenged in the form of reduced DPPH-H, when a hydrogen radical combines with a DPPH radical (25). An EO with DPPH radical scavenging activity is beneficial for inhibiting damage to biomolecules (protein, sugars, PUFA, DNA) caused by reactive radical species in susceptible food and biological systems (24).

#### 3.2.2. ABTS radical scavenging activity

The ABTS radical scavenging assay is also usually employed to determine the *in vitro* antioxidant activity of different substrates. The scavenging activity of KGEO at different concentrations for ABTS radicals was determined. The IC_50_ of the EO was determined to be 1.41 ± 0.01 mg/ml (Table 2). The EO exhibited higher activity toward the ABTS radical compared to the DPPH radical. After proton dissociation, the ABTS radical (ABTS+) accepts a single electron and combines with hydrogen (H+) to form stable ABTS, which plays a significant role in free radical scavenging. The main mechanisms in the process of free radical scavenging are proton priority loss and single electron transfer (26). The DPPH radical scavenging assay can only be used in hydrophobic antioxidant systems, while the ABTS radical scavenging assay can be used in hydrophobic and lipophilic systems, which are more common (27).

#### 3.2.3. Hydroxyl radical scavenging activity

Hydroxyl radicals are powerful oxidants that can react with many redox-sensitive elements and organics non-selectively in the environment and damage various biomolecules (28). As a main ROS, hydroxyl radicals can lead to lipid peroxidation or other oxidative damage (29). Different concentrations of KGEO were evaluated. KGEO had hydroxyl radical scavenging activity, with an IC_50_ of 3.09 ± 0.34 mg/ml, as shown in Table 2. KGEO exhibited higher activity toward the DPPH radical and lower activity toward the ABTS radical. Similar to DPPH and ABTS radicals, hydroxyl radical species are neutralized by a hydrogen atom.

#### 3.2.4. Reducing power assay

The ferricyanide reduction method was used to determine the reducing power. The electron-donating activity is a significant mechanism of antioxidant activity, which can be indicated by Fe^3+^ reduction (19). The reducing power was assessed on KGEO. As presented in Table 2, the EC_50_ value for Fe^3+^ reduction of KGEO was 389.38 ± 4.07 mg/ml. This result suggested that KGEO has weak potency to donate electrons to reactive free radicals. During the ferric reducing assay, reducing agents reduced the Fe^3+^−ferricyanide complex to the ferrous form. Thus, Fe^2+^ can be assessed by the density of blue color in the reaction medium at 700 nm (30).

### 3.3. *In vivo* antioxidant activities study

#### 3.3.1. Protective effect of KGEO in the H_2_O_2_-induced oxidative stress zebrafish model

**FIGURE 1:**
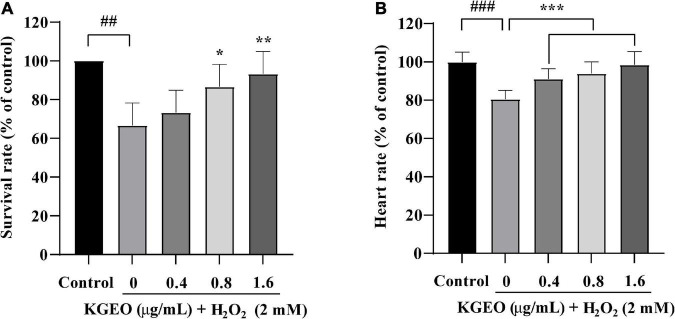
Survival rate and heartbeat rate of H_2_O_2_-induced zebrafish treated with *Kaempferia galanga* essential oil (KGEO). The survival rate **(A)** and the heartbeat **(B)** of zebrafish. The results are expressed as mean ± SD (*n* = 15). ^##^*P* < 0.01, ^###^*P* < 0.001 as compared to the control group. *P < 0.05, **P < 0.01, ***P < 0.001 as compared to the H_2_O_2_-treated group.

The protective effect of KGEO on oxidative stress *in vivo* induced by H_2_O_2_ was established using a zebrafish model. The survival rate of zebrafish embryos treated with different concentrations of KGEO is shown in Figure 1A. After exposure to H_2_O_2_, the survival rate dropped to 66.67% compared to 100.00% of the control group. However, the survival rate increased after preadministration of 0.4, 0.8, and 1.6 μg/ml KGEO, and the rate rose to 73.33, 86.67, and 93.33%, respectively. DMSO (0.32%) as a solvent control had no effect on the survival rate. The heartbeat rate of embryos treated with H_2_O_2_ (2 mM) was decreased to 80.56%, while pretreatment with different concentrations of KGEO (0.4, 0.8, and 1.6 μg/ml) recovered the heart rate to 91.16, 93.94, and 98.48%, respectively (Figure 1B). These results suggested that KGEO alleviated oxidative damage caused by H_2_O_2_.

**FIGURE 2:**
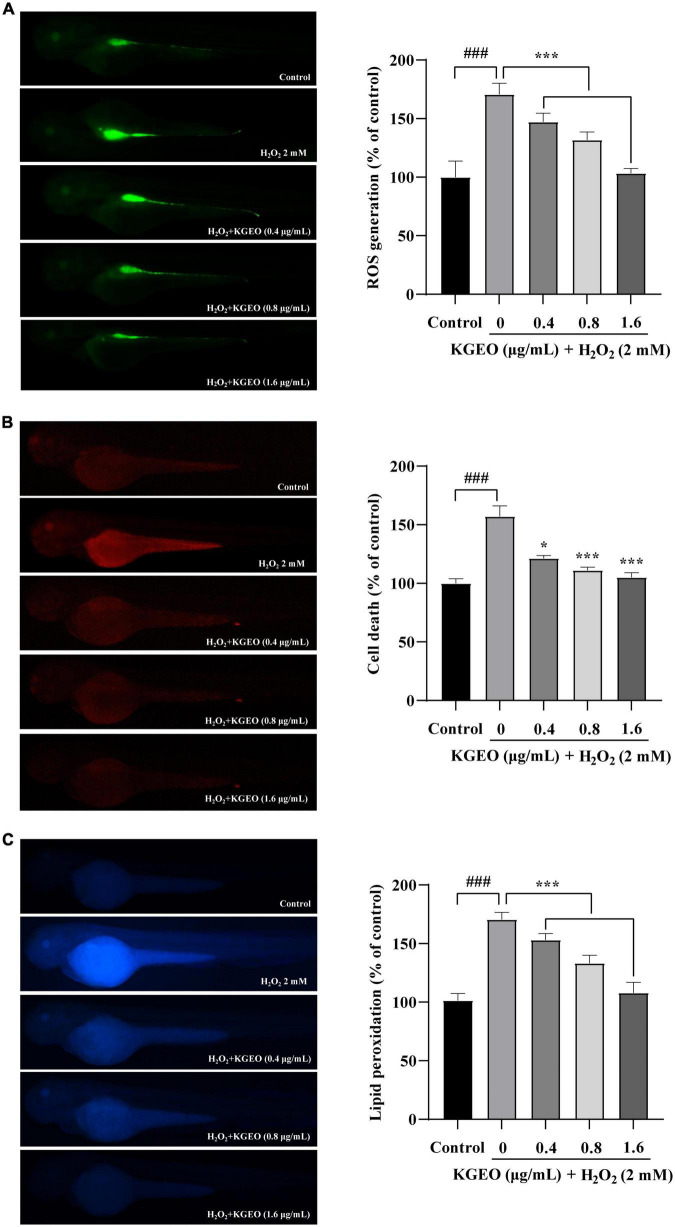
The effects of *Kaempferia galanga* essential oil (KGEO) on H_2_O_2_-induced ROS Production, cell death, and lipid peroxidation in zebrafish. The protective effect of KGEO against H_2_O_2_-induced ROS generation **(A)**, Cell death **(B)**, and lipid peroxidation **(C)**. The relative fluorescence intensities of zebrafish were analyzed using ImageJ software. The data were expressed as the mean ± SD (*n* = 15). ^###^*P* < 0.001 as compared to the control group. *P < 0.05, ***P < 0.001 as compared to the H_2_O_2_-treated group.

The fluorescence images of intracellular ROS generation are shown in Figure 2A. After treatment with H_2_O_2_, the DCF fluorescence intensity was enhanced to 170.63%, with brighter fluorescence compared to the control group, while the fluorescence intensity was reduced up to 103.31% by cotreatment with KGEO.

Acridine orange preferentially stains necrotic and late apoptotic cells, as it is able to permeate cells with disrupted plasma membranes. Fluorescence images showing cell death in zebrafish embryos as assessed by acridine orange staining are shown in Figure 2B. In the H_2_O_2_-treated group, the fluorescence intensity was significantly enhanced to 157.01% compared with the control group. After KGEO was administered, the fluorescence expression was significantly decreased, and the fluorescence value decreased to 105.04% with increasing KGEO concentration.

The fluorescence images for DPPP are shown in Figure 2C, and the fluorescence intensity indicated the degree of lipid peroxidation. H_2_O_2_ significantly induced lipid peroxidation, with the fluorescence intensity increasing to 170.55%, while lipid peroxidation was reduced to 108.00% after KGEO treatment.

The fluorescence intensities of images of ROS generation, cell death, and lipid peroxidation were measured using ImageJ software. The results suggested that KGEO showed protective activity in zebrafish embryos damaged by H_2_O_2_-induced oxidative damage *via* a decrease in ROS and lipid peroxidation and by alleviating cell death.

#### 3.3.2. Effect of KGEO on antioxidant enzyme activities and MDA levels in H_2_O_2_-treated zebrafish embryos

**FIGURE 3:**
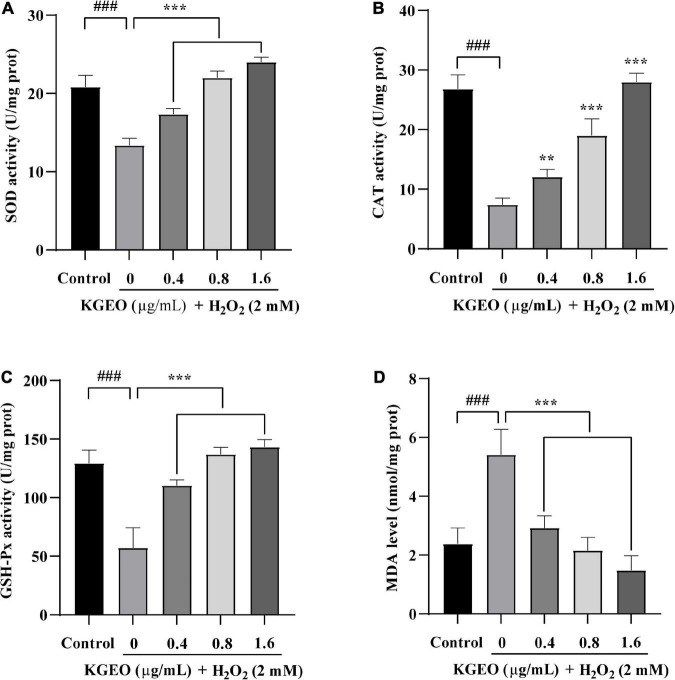
The effects of *Kaempferia galanga* essential oil (KGEO) on levels of malondialdehyde (MDA) and antioxidant enzymes activity in H_2_O_2_-induced zebrafish. Superoxide dismutase (SOD) activity **(A)**, catalase (CAT) activity **(B)**, GSH-Px activity **(C)**, and MDA level **(D)** were determined after incubation. The data are expressed as mean ± SD (*n* = 15). ^###^*P* < 0.001 as compared to the control group. **P < 0.01, ***P < 0.001 as compared to the H_2_O_2_-treated group.

The antioxidant enzyme activities (SOD, CAT, and GSH-Px) and MDA levels were evaluated in H_2_O_2_-induced zebrafish embryos. The results are shown in Figure 3. In the H_2_O_2_-treated group compared to the control group, the activities of CAT, GSH-Px, and SOD in zebrafish embryos were decreased by 3.62−, 2.26−, and 1.56−fold, respectively, and MDA levels were increased by 2.27−fold. However, after treatment with different concentrations of KGEO (0.4, 0.8, and 1.6 μg/ml), the oxidative damage was alleviated in a dose-dependent manner. When treated with KGEO (1.6 μg/ml), the activities of CAT, GSH-Px, and SOD were increased by 3.78−, 2.5−, and 1.80−fold, respectively, and the MDA level showed a decrease of 63.00% compared to the H_2_O_2_-treated group. The results indicated that H_2_O_2_ caused oxidative damage and lipid peroxidation, which were due to the decrease in antioxidant enzyme activities and the increase in the formation of MDA, while KGEO alleviated this damage.

## 4. Discussion

According to the literature, for *K. galanga*, the study of volatile oils has been the focus, especially in China, India, and Malaysia. The results of this study are consistent with those of previous studies, showing that *trans* ethyl p-methoxycinnamate, n-pentadecane, and *trans* ethyl cinnamate were the main constituents in the EO (8). However, after oils were obtained by hydrodistillation (MHD) and traditional hydrodistillation (HD), the main constituents of both oils were similar, but their n-pentadecane (32.80 and 21.60%), *trans* ethyl cinnamate (17.10 and 16.10%) and cyperene (3.40 and 2.00%) contents differed (31). The volatile oil extracted with *n*-hexane by steam distillation was identified as having *trans* ethyl p-methoxycinnamate (38.60%), *trans* ethyl cinnamate (23.20%), 1,8-cineole (11.50%), *trans*-cinnamaldehyde (5.30%), and borneol (5.20%) as the major components, which represented a high content of 1,8−cineole (32). In another study, 39 components were reported from KGEO; among them, *trans* ethyl p-methoxycinnamate, *trans* ethyl cinnamate and *trans* cinnamaldehyde were the main compounds, with contents of 30.60, 26.80, and 11.50%, respectively, which represented a high content of *trans* cinnamaldehyde (33). 1,8-cineole and *trans* cinnamaldehyde were not detected in this oil, which may be related to geography, climate conditions and postharvest processing, and the applied extraction methods also influenced the chemical profile. In addition, the relative percentages of major compounds were also affected by these factors.

Generally, the results of the *in vitro* antioxidant tests described above indicated that KGEO’s scavenging ability for DPPH, ABTS, hydroxyl radicals and reducing power exhibited a concentration-dependent manner comparable to the reference standard, ascorbic acid. Antioxidants can prevent or reduce damaging effects from free radicals in the human body by reacting with free radicals and neutralizing them (34). Moreover, the antioxidant activity of KGEO may be related to different mechanisms because of the chemical complexity of the oil.

Herbs and spices have a long history of being used to improve sensory and flavor characteristics. Many studies have explored the antioxidant activity of EOs and many herbs for addition to various foods. The vapor phase of EOs has bioactivity, which makes them useful for protecting commodities (35).

The zebrafish is a popular *in vivo* model that is widely used for modern bioactivity research and is characterized by easy handling, transparent embryos, and a short generation time. In previous studies, zebrafish stimulated with H_2_O_2_ have been used successfully to estimate antioxidant activity (36–37). As a result, H_2_O_2_-stimulated zebrafish were selected to evaluate the *in vivo* antioxidant effect of KGEO as an *in vivo* model. *In vivo* zebrafish studies indicated that KGEO alleviated oxidative stress damage and reversed the ROS generation, cell death and lipid peroxidation induced by H_2_O_2_.

The uncontrolled generation of free radicals and their damage to antioxidant defensive mechanisms cause superfluous ROS and induce oxidative damage (38). SOD, CAT, and GSH-Px are regarded as important defense systems against oxidative stress. O_2_^–^ is converted to H_2_O_2_ by SOD and then is detoxified to H_2_O and O_2_ either by CAT or GSH-Px. SOD also inhibits lipid peroxidation by terminating the chain reaction. MDA is an indicator of lipid peroxidation, which is caused by redundant ROS (39). This study showed that KGEO could alleviate this damage caused by H_2_O_2_ by increasing the activities of antioxidant enzymes and downregulating the formation of MDA in zebrafish.

## 5. Conclusion

The chemical study of KGEO showed that it contains monoterpenoids, sesquiterpenoids, phenylpropanoids, hydrocarbons, and aliphatic acid esters. Among them, *trans* ethyl p-methoxycinnamate, *trans* ethyl cinnamate, and n-pentadecane were determined to be the major components. The antioxidant activity tests *in vitro* and *in vivo* showed that KGEO could effectively eliminate different free radicals and protect zebrafish embryos against H_2_O_2_-induced oxidative stress. These findings suggested that KGEO had good antioxidant properties. It laid a solid foundation for further research to explain the underlying mechanisms and signaling pathways for the protective effects of KGEO, and is promising for development as a functional ingredient in the pharmaceutical, food and cosmetic industries.

## Data availability statement

The original contributions presented in this study are included in this article/supplementary material, further inquiries can be directed to the corresponding author.

## Ethics statement

The animal study was reviewed and approved by the Guangdong Laboratory Animals Monitoring Institute IACUC.

## Author contributions

S-YW carried out the main experiments, analyzed the data, and wrote the manuscript. LC contributed to the zebrafish experiments. NY performed some of the *in vitro* antioxidant activity tests. F-FX and Y-SW contributed to the revision of the manuscript. BL designed the research plan and conceived the project. All authors contributed to the article and approved the submitted version.
